# A Simple and Inexpensive Stereotactic Guidance Frame for MRI-Guided Brain Biopsy in Canines

**DOI:** 10.1155/2014/139535

**Published:** 2014-05-18

**Authors:** Alexander D. Squires, Yabiao Gao, Sean F. Taylor, Marc Kent, Zion Tsz Ho Tse

**Affiliations:** ^1^College of Engineering, The University of Georgia, Athens, GA 30602, USA; ^2^College of Veterinary Medicine, The University of Georgia, Athens, GA 30602, USA

## Abstract

A magnetic resonance imaging (MRI) guided stereotactic system was developed to provide veterinarians a method to accomplish minimally invasive stereotactic brain biopsies and procedures involving the cerebrum in canines. While MR-guided procedures are prevalent for humans, they are less common in animal practices. The system was designed to minimize fabrication costs in an effort to make such procedures more accessible in the veterinary field. A frame constrained the head without the need for punctures and supported registration and guidance attachments. Location data for registration and relevant structures were selected by the clinician, and a reverse kinematic analysis program generated the settings of the stereotactic arch to guide a needle to the desired location. Phantom experiments and three cadaver trials showed an average targeting error of <3 mm using the system.

## 1. Introduction

Magnetic resonance imaging (MRI) is widely used in medical research and practice due to its ability to peer inside biological organisms with superior image quality, providing high-quality soft tissue imaging while not exposing patients to potentially ionizing radiation or contrast agents [1]. Advances in MRI technology have led to numerous studies and the development of MR-guided treatment techniques [2]. Of particular interest here is the prevalence of MR-guided brain biopsy procedures [3–14] as well as similar procedures performed via computed tomography (CT) [15–18].

Utilizing MRI to perform brain biopsies in humans is common, but in canine subjects tumor diagnosis is most commonly performed postmortem. Naturally, such timing does not help the patient, and access to tumors in vivo is desirable. Image-based diagnosis by itself provides less certainty, and open-skull biopsies require a sizable amount of tissue and bone to be damaged or removed; both cases have their drawbacks [17]. Stereotactic procedures can be much superior, thanks to their precise targeting abilities and the small size of holes in tissue and bone which are required, causing tissue to be minimally damaged.

Inside an MR suite, use of certain materials will degrade image quality and/or endanger the safety of a patient [19]. For this reason, any device which operates on electromagnetic principles (such as common electric motors or relays) is not compatible with the MR environment [20]. Thus, actuation of devices inside the MR suite must be powered using other means, often via pneumatics or piezoceramics [21]. Additionally, any ferro-/paramagnetic materials are banned for safety reasons [19, 20, 22]. Plastics are frequently used for this reason; certain nonmagnetic metals such as aluminum, brassm, or titanium can also be used but run the risk of causing interference with image acquisition and generating artifacts [22–24]. Electrical circuits must be shielded to prevent interference in both directions [1].

Previously, researchers have developed various stereotactic brain biopsy platforms for animal subjects [25]. While previous procedures enabled high-precision stereotaxy, there is plenty of room for optimization. One issue is that some current techniques are based on external markers (skull fiducials). These markers are related to an MRI visualized intracranial target. Depending on MRI slice thickness and the size and shape of the markers, this technique may cause some distortion and inaccuracy during MR imaging and identification of the markers [26]. Another problem can be that coordinates are calculated in 2D space on an MRI viewer screen, while failing to consider 3D reconstruction data or automatic target calculation. Another potential issue is that the trajectory can be constrained to a straight vertical approach, with no angled or oblique targeting in the dorsal or transverse planes available due to their need for more robust and complex calculations requiring greater information than that which is provided in 2D space. Vertical trajectories may restrict the surgeon to unhelpful and inappropriate trajectories. To solve these problems, the currently existing Leskell system, an arch-based stereotactic system for isocentric stereotaxy and 3D coordinate calculations relative to stereotactic frame integrated with fiducial markers, could be implemented. The Leskell system is already clinically available [27]. While there has been some evidence to suggest that free-hand techniques performed under MRI guidance may be the equal or superior to stereotactic techniques [28, 29], the surgeons working with the project expressed a desire for the use of a stereotactic frame, thus causing that avenue to be pursued. Research has demonstrated that frame-based stereotactic systems are more accurate than frameless systems; thus a frame-based method was explored for the construction of the device [30].

This study aimed to integrate fiducial marker* Z*-frames inside the scanner with a modified Leskell stereotactic arch used outside the magnetic field to enable arch-based isocentric stereotaxy. The request for the construction of this device came from the neurosurgical unit of the University of Georgia Veterinary School. Following an MRI scan to locate brain tumors and determine operability, an open skull biopsy is performed to obtain a sample. Desiring a less invasive method of surgery, the developed system took inspiration from previous research and developed a low-cost system (in terms of additional costs to the existing procedure) to integrate with current operational procedures. Given an average tumor diameter of 10 mm to biopsy, the surgeons requested less than 3 mm of error when targeting the center of the tumor.

## 2. Materials and Methods

### 2.1. Overview

Previous studies have been conducted to develop stereotactic cerebral interventions or MRI-compatible guidance frames, including several which implement* Z*-frame approaches and 3-point head fixations [11–14, 25–27, 31–37]. We combined both fields, developing an MRI-guided device to assist veterinarians in executing minimally invasive brain biopsies and other cerebral procedures.

The design consists of a primary frame with two sets of attachments; one for imaging and the other for needle placement. The frame's cross-section is U-shaped in order to maintain sufficient strength and stiffness, contain a canine's head, and fit inside a standard MRI head coil.

The* Z*-frame attachments contain tubes of Vitamin E oil acting as a localizing agent (to replace fiducials) to facilitate image registration. The geometry of the markers ensures that either a dorsal or transverse plane will contain 6 points where the plane intersects the tubes. A LabView program (National Instruments, Austin, Texas) was written to use location data (*X*,* Y*, and* Z* coordinates) from these points and create a mapping of the scanner's coordinate system onto the coordinate system of the frame.

Following image acquisition, the veterinarian determined the location of structure(s) to be targeted. The relevant coordinates from the scanner were mapped onto the frame's coordinate system using the developed transform. After transformation, the relevant coordinates were fed into a second LabView program which performed reverse kinematics calculations to generate the adjustments on the stereotactic arch which enable the point of a biopsy needle to arrive at the target point.

After completing the necessary imaging, the* Z*-frame attachments could be removed outside the MRI suite and replaced with the stereotactic arch and its supports. Once attached, the arch would be adjusted following the parameters established by the program, providing a precise guide for the clinician to perform their procedures.

By utilizing a simple frame, a commonplace material for a contrast agent, and a surplus stereotactic arch, the cost of materials for the project was minimized. An outline of pricing for the materials used in the final system is shown in Table 1.

### 2.2. Mechanical Design

**Figure 1:**
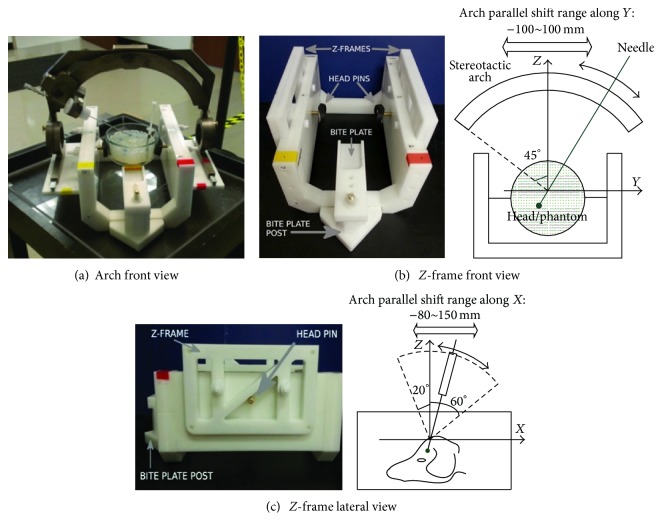
(a) Stereotactic arch and frame assembled for needle guidance. Design (left) and workspace (right) of the stereotactic guidance frame in front (b) and lateral (c) views.

The body of the device (Figure 1(a)) consists of four sets of components: the primary frame, the* Z*-frames, the arch support attachments, the stereotactic arch, and a 14-gauge biopsy needle. The primary frame consists of Delrin U-shaped end pieces with Delrin lateral supports (Figures 1(b) and 1(c)). The supports constrain the attachment pieces. Zygomatic arch supports are located in the center of the lateral supports and consist of a rubber sheet over an ABS concave support, which is attached to a brass threaded rod. A post to support the bite plate (both Delrin) is attached to one of the end pieces. The support structures are independent from the attachment pieces, ensuring patients do not shift during transition between attachments. These supports are noninvasive, eliminating the need for bone screws to support fiducial markers or a stereotactic device. The frame was constructed to conform to the space within the head coil used by the veterinary neurosurgeon. The frame secures head widths between 7 and 20 cm. The frame's design could be widened to support the heads of larger breeds as well, in conjunction with a body coil.

The* Z*-frames and arch attachments were also manufactured from Delrin. Internal channels in the* Z*-frames contain removable tubes filled with Vitamin E oil. The arch supports have aluminum rails to mount the stereotactic arch. Both sets of attachments attach to the frame via nylon screws and nuts and are capable of being changed out without disturbing patient fixation.

The stereotactic arch has 5 degrees of freedom. The arch slides along the rails of the arch support frame (*X*), rotates about an axis drawn between the rails (rotates around* Y*), slides along that same axis (*Y*), and rotates about an axis parallel and central to the two rails (rotates around* X*). Additionally, the support frames can be set to one of two vertical positions and the needle depth adjusted with a stopper (both affect* Z* position).

### 2.3. *Z*-Frame Design

**Figure 2:**
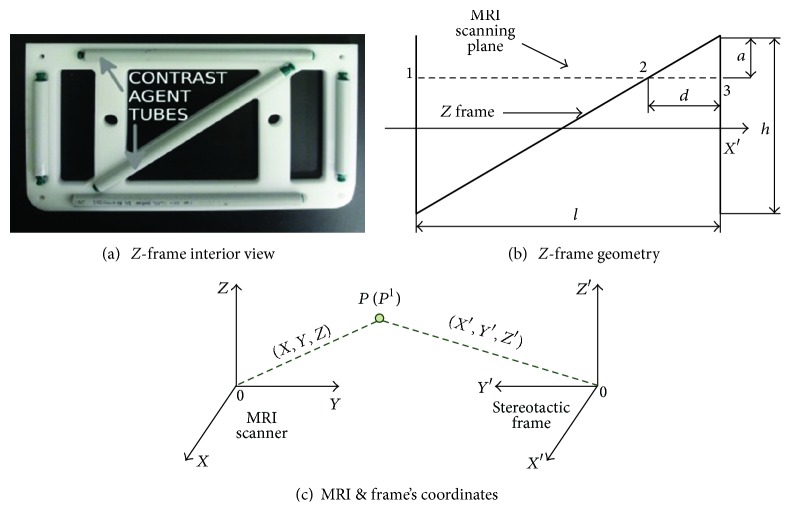
(a)* Z*-frame design. (b) Calculation of 6 points to* X*,* Y*, and* Z* coordinate in guidance frame and (c) MRI-frame registration.

The* Z*-frames each contain five tubes filled with an image contrast agent (Figure 2(a)). Four tubes form a rectangle, while the fifth lies along a diagonal. When attached to the primary frame, the diagonals form an* X* when viewed laterally (Figure 2(b)). This configuration allows either a dorsal or transverse image slice to be used to develop a coordinate system for the frame and dog. The opposing diagonals generate a unique solution for any possible plane which intersects the* Z*-frames, allowing the true position of the frame to be known.

### 2.4. Image Registration

**Figure 3:**
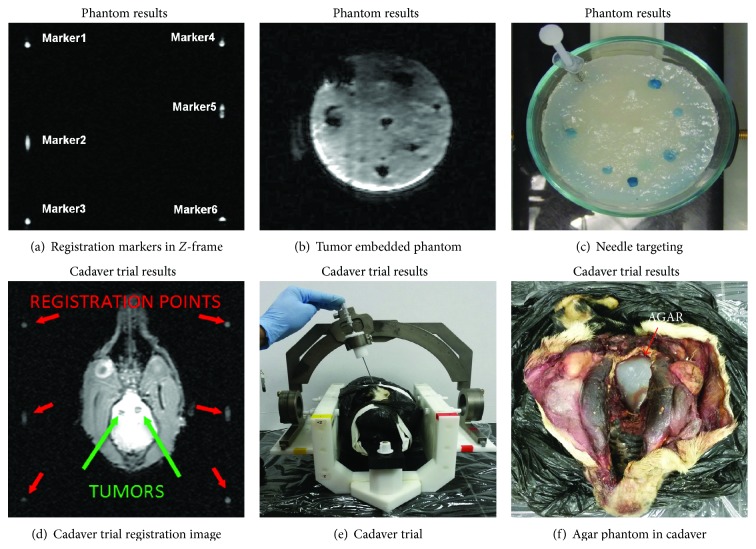
MRI images of (a) the* Z*-frame markers for registration and (b) of the target tumor target in the agar phantom. (c) Photo of agar phantom. (d) Registration image from cadaver trial and (e) performing the cadaver biopsy. (f) Agar embedded in the skull.

A mapping between the MRI coordinate system (*P*) and the stereotactic frame coordinate system (*P* ^1^) is required to perform kinematic calculations. We used known physical relationships of the* Z*-frames (Figure 2(a)) in the stereotactic frame coordinate system to calculate a transformation matrix between the two coordinate systems (Figure 2(c)). Markers 1, 3, 4, and 6 in Figure 3(a) were used to calculate a 4 × 4 transformation matrix *T* using the formula *P* = *T* · *P* ^1^ [38] in a Matlab program (MathWorks, Natick, Massachusetts) embedded into the LabView interface. Since an arbitrary frontal plane is imaged to obtain registration points, the relationship between the scanner* Z*-coordinate and frame* Z*-coordinate is initially unknown; it is calculated using distances between registration points in the frontal plane image.  Figure 3(b) shows the relationships used when calculating the* Z*-coordinate of the base frame. The* Z*-coordinate in the frame coordinate system is calculated as

(1) $$\begin{array}{c} z=\frac{\left(l-2d\right)h}{2l}, \end{array}$$

where the lengths are taken from the registration image; *d* is the distance between marker 2 and 3, *h* is the length of the* Z*-frame in* Z*-axis of stereotactic coordinate system, and *l* is the length of the* Z*-frame in* X*-axis of stereotactic coordinate system. After the mapping process was finished, the stereotactic coordinates can be converted from their MR coordinates via inverse transformation. Assuming the coordinate matrix of a target point is *C* in the MR scanner coordinate system and its corresponding coordinate in stereotactic system is *C* ^1^, then *C* ^1^ = *T* ^−1^ · *C*. The inverse kinematics necessary to position the stereotactic arch are processed in a second LabView interface; an operator can adjust the trajectory and observe the required settings of the stereotactic frame to hit the target “tumor.”

## 3. Results and Discussion

### 3.1. Experimental Setup in MRI

Phantom tests (Figure 2(c)) and preliminary cadaver studies (Figure 3(e)) to confirm the safety and precision of the system were performed in a 3T GE MRI scanner. The phantoms used in the experiments were made from agar gel containing tumor representations made of 5 mm to 10 mm diameter balls of putty (Figure 3(c)) which displayed as a dark spot on a lighter background in the MRI images (Figures 3(a) and 3(d)). For the cadaver studies, the cerebrum was removed and replaced with the phantom material (Figure 3(f)).

For the MRI tests, a vial of image contrast agent was placed in the phantom in the +*X*, +*Y* quadrant of the primary frame to provide an easily distinguishable reference point. The phantom was placed in the frame and fixed via the zygomatic arch supports and a modified bite plate. The* Z*-frame attachments were placed on the frame, and the system was scanned with a gradient echo sequence (TR = 200, flip angle = 30, slice thickness = 3 mm, pixel size = 1 mm × 1 mm, number of slices = 15, FOV = 300 mm × 300 mm, BW = 130 Hz/pixel). Signal-to-noise ratio reductions were under 2% while the system was in the scanner. After acquiring images containing the points necessary for registration (Figure 3(a)) and the locations of the “tumors” (Figure 3(b)), the system was removed from the MRI.

Following the removal from the scanner room, the* Z*-frames were replaced by the arch and its support attachments. The settings on the arch to target a particular “tumor” were processed by the LabView programs; the arch was then adjusted to match those settings by an operator. Finally, a stopper that was placed on a biopsy needle to halt it progress at the indicated depth was added. The biopsy needle was then inserted into the arch and then into the phantom.

### 3.2. System Precision

The sum of squared errors in the registration process ranged from 2.40 mm to 3.50 mm in our experiment. Errors were likely caused by* Z*-frame placement error in the MRI scanner, slight inaccuracies in base frame measurements for the stereotactic frame coordinate system, and measurement errors when determining registration marker coordinates in the MRI. We visually determined the center of the tumor on screen to serve as the target, which may not be the most accurate value. The result would improve with an algorithm calculating the center of target utilizing multiple images.

Distances between needle tip placement and the centers of target tumors were quantified by segmenting the phantom. The error while targeting the phantom was <3 mm in all trials, which met the minimum requirements of the neurosurgeons. Slight errors in the registration process influence the error in targeting the tumor, because the registration matrix converts the tumor's MRI coordinates to the frame's coordinate system. Notably, the most recent cadaver trial had a mean targeting error of 2.5 mm with a sum of squared errors in the registration process of 2.43 mm, indicating that registration errors are the major contributing factor to targeting error. The time required for each task in the process is shown in Table 2. Errors stem from tissue deformation and needle flexing during insertion. These errors present themselves in comparable MRI-guided targeting devices, with mean targeting errors ranging from 1.79 mm to 4.4 mm [14, 39, 40]. Similar CT procedures resulted in mean targeting errors between 2.8 mm and 3.6 mm [15–17].

## 4. Conclusion

A stereotactic device for MRI-guided biopsy was designed and fabricated. The device was designed to be small enough to fit into a head coil, with a setup time in the MRI scanner of less than 5 minutes. The device was tested in a GE 3T MRI scanner using a custom made agar phantom with embedded simulated tumors. The time for manual detection and registration of the 6 marker points in the* Z*-frames was less than 10 minutes.

A biopsy targeting error of <3 mm was measured, which outperforms the goal of set by our veterinary neurosurgeon for targeting 10 mm diameter tumors. Programs with a graphical user interface were developed for the computation of the coordinate system after registration, stereotactic arch movement, and needle trajectory planning. Computation time was less than 3 minutes per tumor target and the manipulation of the stereotactic arch to target a tumor took less than 1 minute, for a total targeting time of less than 4 minutes per target. Our experiment demonstrated that all tumors (6 out of 6) were targeted and hit by the needle with an average accuracy of 2 mm.

## 5. Future Work

Ongoing work aims to improve registration point selection through the use of feature-based image registration techniques. The system will be tested in a cohort of swine and canine cadavers to verify the functionality and accuracy of the system. After verification, the system will be used for the testing and treatment of live patients.

## Figures and Tables

**Table 1:** Materials costs.

Delrin	$175
Aluminum	$20
Stereotactic arch	$600
Contrast agent	$10
Assorted hardware	$85
Fabrication shop labor	$500

Total	$1410

**Table 2:** Procedure time summary.

Task	Time required
Device setup in MRI scanner	2 minutes
Localizer scan	3 minutes
Manual detection of image markers	5 minutes
Registration	5 minutes
Inverse kinematics	3 minutes
Manual joint movement	1 minute

Total	19 minutes
